# Getting it right is better than being right, right?

**DOI:** 10.1186/s42155-023-00420-8

**Published:** 2024-01-03

**Authors:** Jim A. Reekers

**Affiliations:** Amsterdam UMC, University of Amsterdam, Meibergdreef 9, Amsterdam, The Netherlands

The dilemma of interventional radiology is that being right does not automatically translate into getting it right. I found out, amid the turmoil following the publication of my book *The Medical Omerta* (published in Dutch) that there was a significant interest not only on social media but also in newspapers, radio, and television about uterine fibroid embolization. It seemed that all our efforts during the last 15 years to give this topic more public attention had completely failed, as the message about UFE being a proven alternative to hysterectomy came totally out of the blue for many women. A paper in 2019 about implementation of UFE in the Netherlands, less than 6%, was already a predictor of the bad news [1]. Here there is some similarity with other women-related IR procedure, which we have highlighted in our CVIR Endovascular special issue on women’s health [2].

My personal story about the failure to get UFE implemented in the Netherlands after our EMMY trial is only one chapter out of fourteen in my book, but the discussions about this chapter overshadowed all the other chapters. After the release of my book, women posted their personal, and always bad, experiences with hysterectomy on social media. It was interesting to see that the focus was on two aspects of the UFE saga. First was the complete absence of any information on UFE by the gynaecologists during consultation and second was the importance for many women to preserve their uterus to maintain fertility and as a crucial part of their femininity [3]. The fact that gynaecologists do not tell patients about UFE is well known worldwide and supported by many papers [4], but the high focus for women to preserve their uterus as a crucial part of their femininity came also to me as a total surprise. During the aftermath of my book release, the discussions with gynaecologists were mostly personal attacks on me in newspapers and on social media. One gynaecologist wrote in a newspaper interview that women were always very relieved, in his personal experience, to have their uterus removed which has given them so much trouble. He said that more than 80% of women in his practice choose to have their uterus removed instead of undergoing UFE. Of course, this was his personal male experience without any science to back it up.

What we have been showing with level 1 evidence and a 10-year follow-up is that UFE is a true alternative to hysterectomy for the endpoint quality of life. This is what I mean with Being Right based on scientific data, but unfortunately, we have not been able to Get it Right for the patients. In most European countries the number of UFE is between 0 and 6%, at the most. We have been following the endpoints of the gynaecologists by focusing on avoiding major surgery and shorter hospital stay, which is countered by gynaecologists with the argument that laparoscopic hysterectomy is also not major surgery and also requires only a one-day hospital stay. But we have completely overlooked the major benefits of UFE, as expressed by many women, which are fertility and the preservation of their uterus. There is a very interesting paper which focuses on the mental and physical problems experienced by women after hysterectomy, which tells the real and still heavily denied truth – especially by gynaecologists – about the period after hysterectomy [5].

What can we, as IR, learn from this experience? First and foremost, we should not end up in a discussion solely comparing our IR results with results of other medical specialties without highlighting the unique features that most IR treatments have. Secondly, we should try to find out where IR really matters to patients. This means that we should organize patient audits to not only be right but also to get it right. And then promote those endpoints that make a real difference in QOL for patients. These can be both physical but also mental. I see the same thing happening now with prostate embolization (PAE) where again we are in competition with urologists on endpoints like post-procedural PSA dynamics. But what matters most to men are the complications of surgical treatment of BPH like bleeding, ureteral orifice injury, bladder neck injury, rectal injury, TURP syndrome, bladder neck contractures, urethral stricture disease, refractory OAB symptoms, and retrograde ejaculation. Overactive bladder symptoms (OAB) are rarely discussed with the patient but have a major impact on the QOL. OAB is characterized by a group of four symptoms: urgency, urinary frequency, nocturia, and urge incontinence [6]. None of these complications are known from prostate embolization. By the same token as we should advertise UFE with ‘Preservation of the uterus,’ we should promote PAE as ‘A life without diapers!’

Without going into detail there are many more IR procedures with unique QOL selling points that really make a difference for patients. In interventional oncology, QOL often prevails, at least from the patient’s point of view, a short gain in survival and with MSK embolization the QOL gain is also evident. With every IR procedure we perform, we have to start thinking from a patient QOL perspective - combining this with an IR perspective and forget about the competition with other medical specialties for only medical physiological parameters.

In conclusion, by emphasising the unique features of IR, especially QOL data that matter to patients in daily life, we can underline the unique selling points of IR. We should not fall into the trap of competing with the endpoints of other medical specialties. And we should not be afraid to create a public debate and even turmoil on these issues because, by the end of the day, we will be supported by our patients.

I wish you all a good 2024 and I hope also next year to receive your scientific papers for CVIR Endovascular.
